# The genome sequence of the Pebble Hook-tip, *Drepana falcataria* (Linnaeus, 1758)

**DOI:** 10.12688/wellcomeopenres.19213.1

**Published:** 2023-05-10

**Authors:** Douglas Boyes, Owen T. Lewis, University of Oxford and Wytham Woods Genome Acquisition Lab, Darwin Tree of Life Barcoding collective, Wellcome Sanger Institute Tree of Life programme, Wellcome Sanger Institute Scientific Operations: DNA Pipelines collective, Tree of Life Core Informatics collective, Darwin Tree of Life Consortium

**Affiliations:** 1 UK Centre for Ecology & Hydrology, Wallingford, England, UK; 2 Department of Biology, University of Oxford, Oxford, England, UK

**Keywords:** Drepana falcataria, the Pebble Hook-tip, genome sequence, chromosomal, Lepidoptera

## Abstract

We present a genome assembly from an individual male *Drepana falcataria* (the Pebble Hook-tip; Arthropoda; Insecta; Lepidoptera; Drepanidae). The genome sequence is 326.7 megabases in span. The whole assembly is scaffolded into 31 chromosomal pseudomolecules, including the assembled Z sex chromosome. The mitochondrial genome has also been assembled and is 15.4 kilobases in length.

## Species taxonomy

Eukaryota; Metazoa; Ecdysozoa; Arthropoda; Hexapoda; Insecta; Pterygota; Neoptera; Endopterygota; Lepidoptera; Glossata; Ditrysia; Drepanoidea; Drepanidae; Drepaninae; *Drepana*; *Drepana falcataria* (Linnaeus, 1758) (NCBI:txid104428).

## Background

*Drepana falcataria*, the Pebble Hook-tip, is the largest and commonest of the hook-tip moths (family Drepanidae) in Britain and Ireland. The English name of these moths reflects the distinctive hooked appearance of their forewings. In the case of *D. falcataria*, the pebble-like central spot on the forewing, combined with a purplish-brown blotch near the wing tip, distinguish this species from the other six Drepanidae species recorded in Britain and Ireland ( Waring *et al.*, 2017).

This species occurs in woodlands, gardens, heathland and similar habitats across much of Britain and more locally in Ireland ( Randle *et al.*, 2019; Waring *et al.*, 2017). It has expanded its distribution significantly since 1970, but has also declined in abundance ( Randle *et al.*, 2019). *Drepana falcataria* has a wide distribution across western and central Europe and Siberia ( GBIF Secretariat, 2022).

The main larval foodplants of *D. falcataria* are birches ( *Betula* spp.) and alder ( *Alnus glutinosa*) ( Henwood *et al.*, 2020). It over-winters as a pupa, and adults fly at night from late April to June, with a second brood which peaks in August ( Randle *et al.*, 2019). The spring generation now appears significantly earlier in the year than it did in the 1970s ( Randle *et al.*, 2019). In the north, this species has a single annual generation, and in the northern half of Scotland occurs as a distinct subspecies, *scotica*, which has a paler ground colour ( Waring *et al.*, 2017).

A genome sequence for *Drepana falcataria* will contribute to a growing data set of resources for understanding lepidopteran biology. The genome of *Drepana falcataria* was sequenced as part of the Darwin Tree of Life Project, a collaborative effort to sequence all named eukaryotic species in the Atlantic Archipelago of Britain and Ireland. Here we present a chromosomally complete genome sequence for *Drepana falcataria*, based on one male specimen from Wytham Woods, Oxfordshire, UK. 

## Genome sequence report

**Figure 1.**
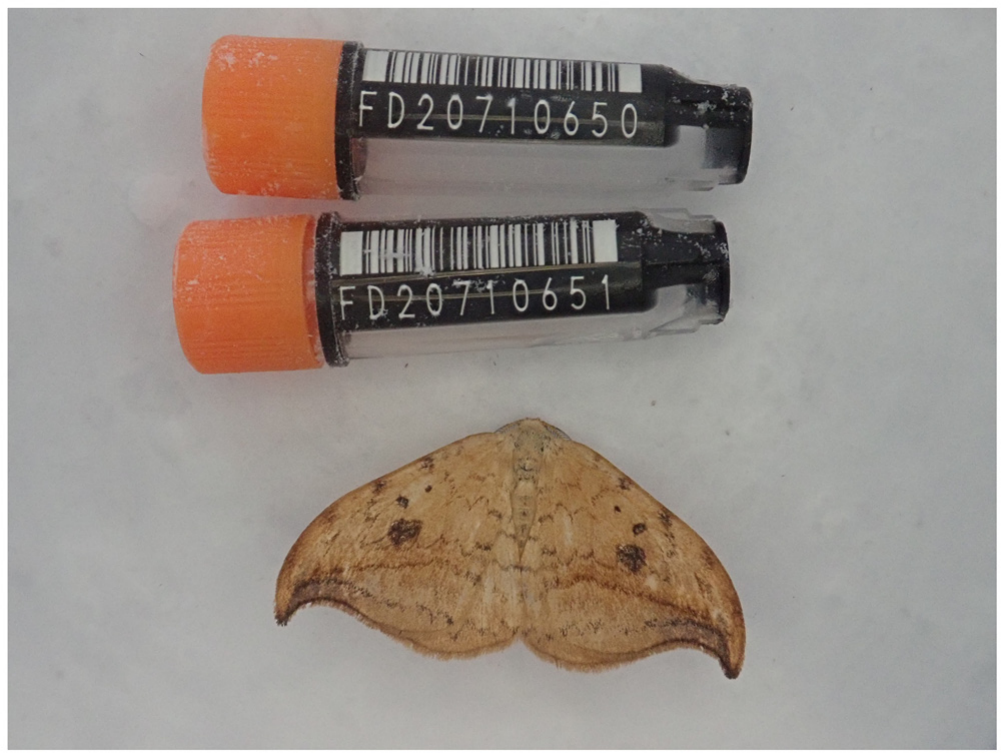
Photograph of the *Drepana falcataria* (ilDreFalc1) specimen used for genome sequencing.

The genome was sequenced from one male *Drepana falcataria* ( Figure 1) collected from Wytham Woods, Oxfordshire, UK (latitude 51.77, longitude –1.34). A total of 73-fold coverage in Pacific Biosciences single-molecule HiFi long reads was generated. Primary assembly contigs were scaffolded with chromosome conformation Hi-C data. Manual curation corrected two missing joins or misjoins.

**Figure 2.**
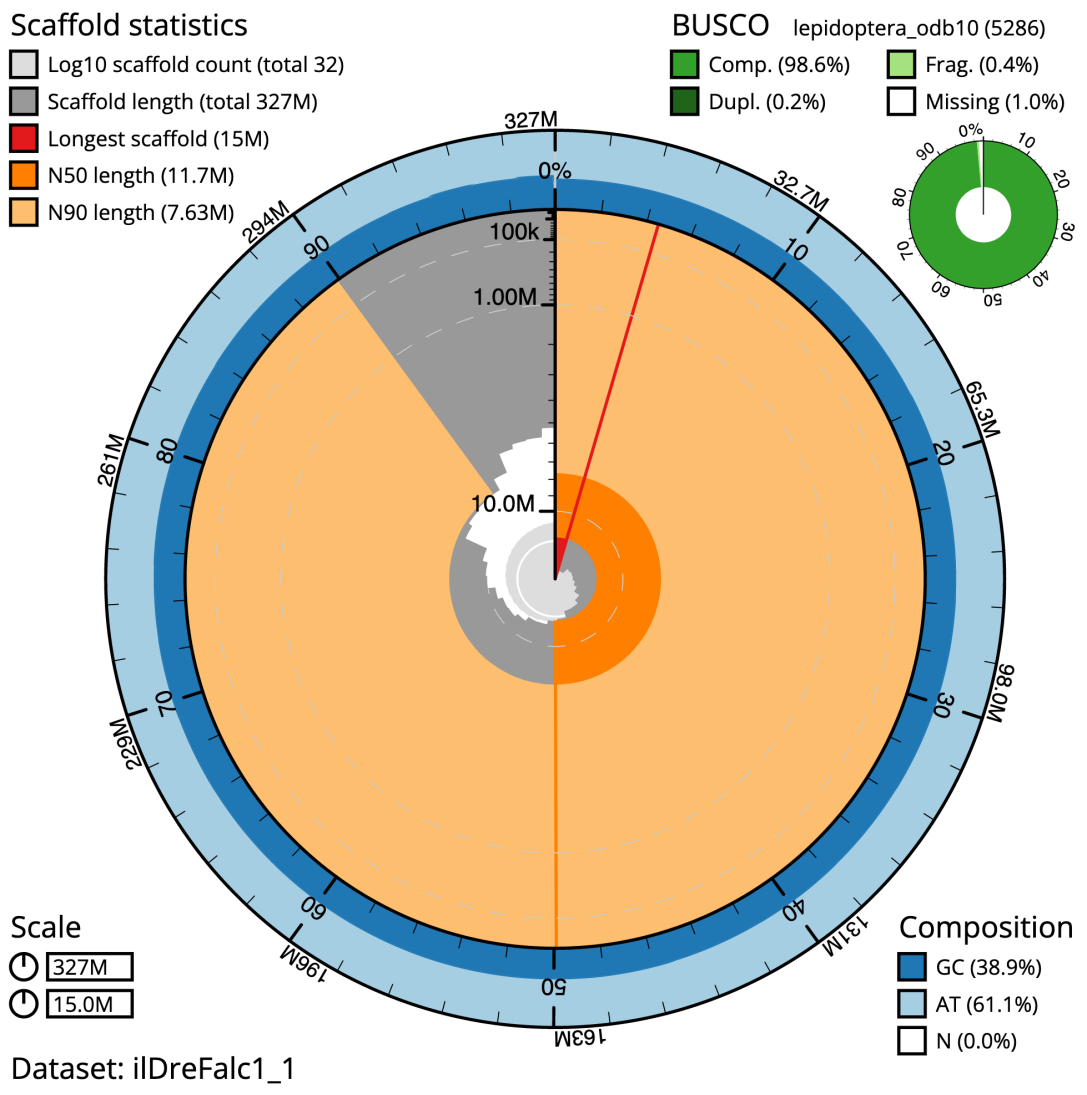
Genome assembly of *Drepana falcataria*, ilDreFalc1.1: metrics. The BlobToolKit Snailplot shows N50 metrics and BUSCO gene completeness. The main plot is divided into 1,000 size-ordered bins around the circumference with each bin representing 0.1% of the 326,745,212 bp assembly. The distribution of scaffold lengths is shown in dark grey with the plot radius scaled to the longest scaffold present in the assembly (14,982,236 bp, shown in red). Orange and pale-orange arcs show the N50 and N90 scaffold lengths (11,692,006 and 7,634,482 bp), respectively. The pale grey spiral shows the cumulative scaffold count on a log scale with white scale lines showing successive orders of magnitude. The blue and pale-blue area around the outside of the plot shows the distribution of GC, AT and N percentages in the same bins as the inner plot. A summary of complete, fragmented, duplicated and missing BUSCO genes in the lepidoptera_odb10 set is shown in the top right. An interactive version of this figure is available at https://blobtoolkit.genomehubs.org/view/ilDreFalc1_1/dataset/ilDreFalc1_1/snail.

The final assembly has a total length of 326.7 Mb in 31 sequence scaffolds with a scaffold N50 of 11.7 Mb ( Table 1). The whole assembly sequence was assigned to 31 chromosomal-level scaffolds, representing 30 autosomes, and the Z sex chromosome. Chromosome-scale scaffolds confirmed by the Hi-C data are named in order of size ( Figure 2– Figure 5; Table 2). The assembly has a BUSCO v5.3.2 ( Manni *et al.*, 2021) completeness of 98.6% (single 98.4%, duplicated 0.2%) using the lepidoptera_odb10 reference set. While not fully phased, the assembly deposited is of one haplotype. Contigs corresponding to the second haplotype have also been deposited.

**Table 1.** Genome data for *Drepana falcataria*, ilDreFalc1.1.

Project accession data		
Assembly identifier	ilDreFalc1.1	
Species	*Drepana falcataria*	
Specimen	ilDreFalc1	
NCBI taxonomy ID	104428	
BioProject	PRJEB54059	
BioSample ID	SAMEA10979061	
Isolate information	ilDreFalc1; male, head and thorax tissue (genome sequencing and Hi-C scaffolding)	
Assembly metrics *		*Benchmark*
Consensus quality (QV)	67.7	*≥ 50*
*k*-mer completeness	100%	*≥ 95%*
BUSCO **	C:98.6%[S:98.4%,D:0.2%], F:0.4%,M:1.0%,n:5,286	*C ≥ 95%*
Percentage of assembly mapped to chromosomes	100%	*≥ 95%*
Sex chromosomes	Z sex chromosome	*localised homologous pairs*
Organelles	Mitochondrial genome assembled	*complete single alleles*
Raw data accessions		
PacificBiosciences SEQUEL II	ERR9924616	
Hi-C Illumina	ERR9930691	
Genome assembly		
Assembly accession	GCA_945859725.1	
*Accession of alternate haplotype*	CA_945859695.1	
Span (Mb)	326.7	
Number of contigs	34	
Contig N50 length (Mb)	11.6	
Number of scaffolds	31	
Scaffold N50 length (Mb)	11.7	
Longest scaffold (Mb)	15.0	

* Assembly metric benchmarks are adapted from column VGP-2020 of “Table 1: Proposed standards and metrics for defining genome assembly quality” from ( Rhie *et al.*, 2021). ** BUSCO scores based on the lepidoptera_odb10 BUSCO set using v5.3.2. C = complete [S = single copy, D = duplicated], F = fragmented, M = missing, n = number of orthologues in comparison. A full set of BUSCO scores is available at https://blobtoolkit.genomehubs.org/view/ilDreFalc1_1/dataset/ilDreFalc1_1/busco.

**Figure 3.**
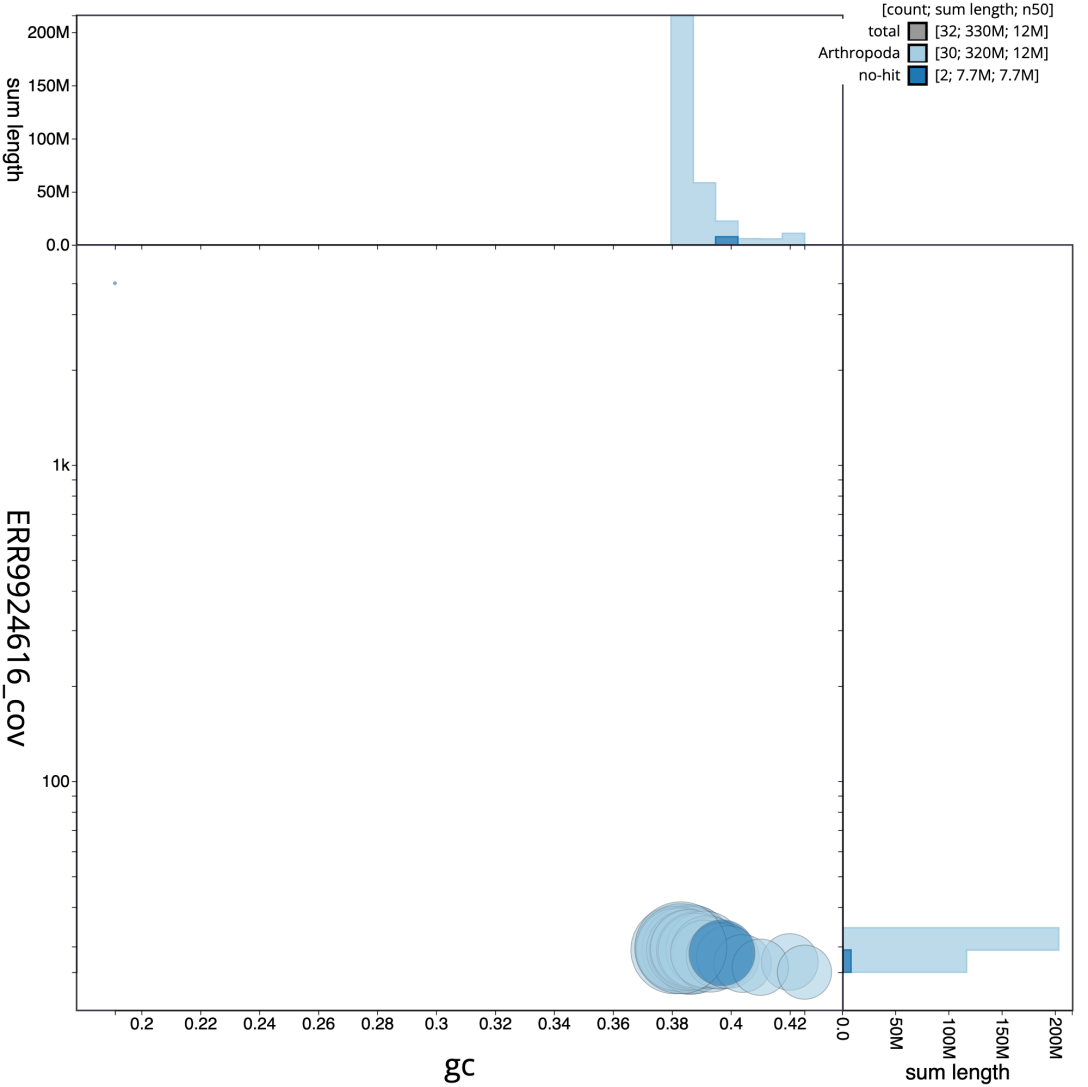
Genome assembly of *Drepana falcataria*, ilDreFalc1.1: GC coverage. BlobToolKit GC-coverage plot. Scaffolds are coloured by phylum. Circles are sized in proportion to scaffold length. Histograms show the distribution of scaffold length sum along each axis. An interactive version of this figure is available at https://blobtoolkit.genomehubs.org/view/ilDreFalc1_1/dataset/ilDreFalc1_1/blob.

**Figure 4.**
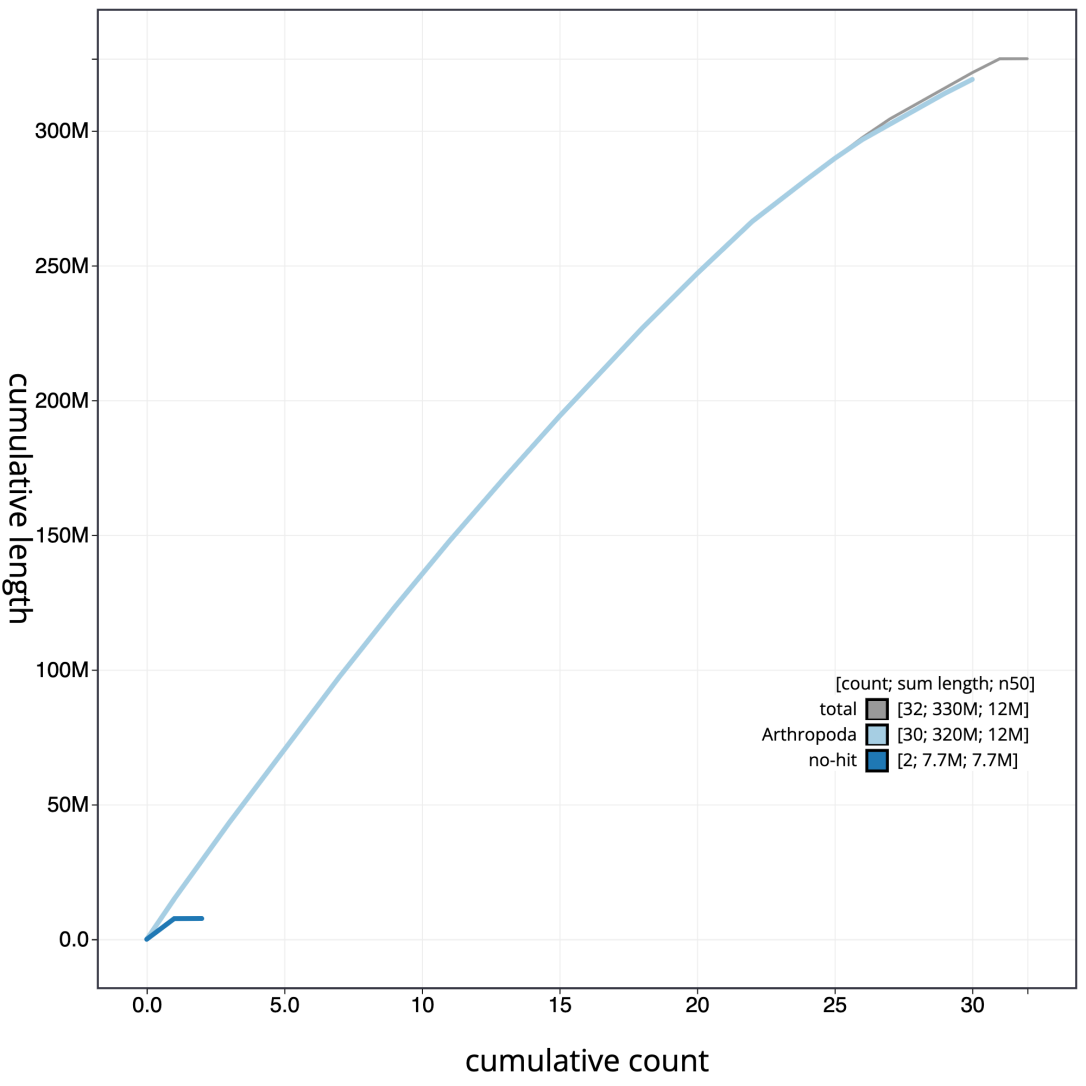
Genome assembly of *Drepana falcataria*, ilDreFalc1.1: cumulative sequence. BlobToolKit cumulative sequence plot. The grey line shows cumulative length for all scaffolds. Coloured lines show cumulative lengths of scaffolds assigned to each phylum using the buscogenes taxrule. An interactive version of this figure is available at https://blobtoolkit.genomehubs.org/view/ilDreFalc1_1/dataset/ilDreFalc1_1/cumulative.

**Figure 5.**
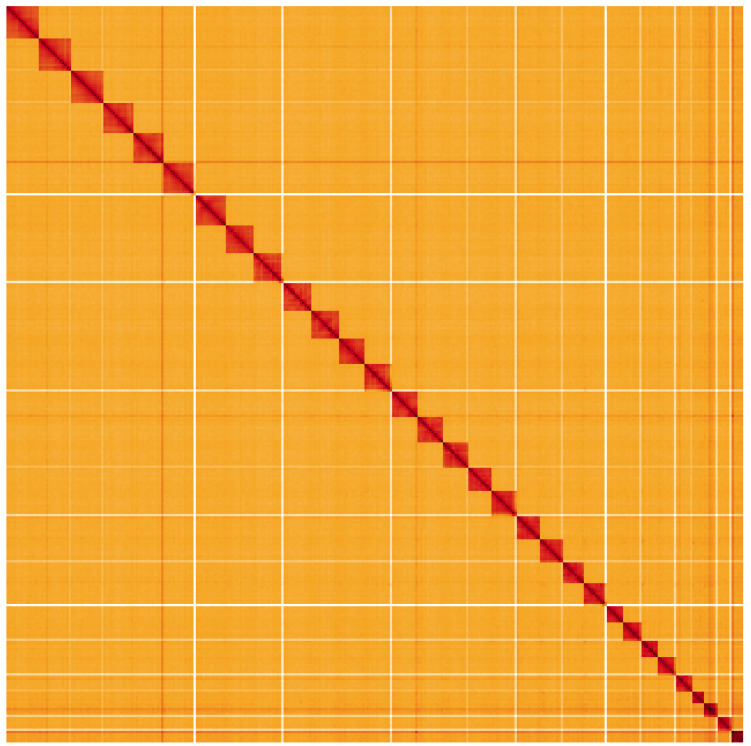
Genome assembly of *Drepana falcataria*, ilDreFalc1.1: Hi-C contact map. Hi-C contact map of the ilDreFalc1.1 assembly, visualised using HiGlass. Chromosomes are shown in order of size from left to right and top to bottom. An interactive version of this figure may be viewed at https://genome-note-higlass.tol.sanger.ac.uk/l/?d=f_ybUAspTj2zY-keVs2uqQ.

**Table 2.** Chromosomal pseudomolecules in the genome assembly of *Drepana falcataria*, ilDreFalc1.

INSDC accession	Chromosome	Size (Mb)	GC%
OX243922.1	1	14.2	38.3
OX243923.1	2	14.15	38.6
OX243924.1	3	13.64	38.1
OX243925.1	4	13.54	38.6
OX243926.1	5	13.5	38.3
OX243927.1	6	13.35	38.6
OX243928.1	7	13.11	38.5
OX243929.1	8	12.64	38.2
OX243930.1	9	12.34	38.4
OX243931.1	10	12.32	38.2
OX243932.1	11	11.84	38.7
OX243933.1	12	11.69	38.6
OX243934.1	13	11.41	38.6
OX243935.1	14	11.38	38.5
OX243936.1	15	11.05	38.6
OX243937.1	16	10.8	38.6
OX243938.1	17	10.75	38.8
OX243939.1	18	10.42	39.1
OX243940.1	19	9.91	38.8
OX243941.1	20	9.82	39.3
OX243942.1	21	9.48	39.2
OX243943.1	22	8.02	39.1
OX243944.1	23	7.77	39.7
OX243945.1	24	7.67	39.7
OX243946.1	25	7.63	39.6
OX243947.1	26	6.96	39.9
OX243948.1	27	5.9	40.4
OX243949.1	28	5.67	42
OX243950.1	29	5.62	41
OX243951.1	30	5.18	42.5
OX243921.1	Z	14.98	38.3
OX243952.1	MT	0.02	18.8

## Methods

### Sample acquisition and nucleic acid extraction

A male *Drepana falcataria* (ilDreFalc1) was collected from Wytham Woods, Oxfordshire (biological vice-county: Berkshire) (latitude 51.77, longitude –1.34) on 24 July 2021. The specimen was taken from grassland habitat by Douglas Boyes (University of Oxford) using a light trap. The specimen was identified by Douglas Boyes and preserved on dry ice.

DNA was extracted at the Tree of Life laboratory, Wellcome Sanger Institute (WSI). The ilDreFalc1 sample was weighed and dissected on dry ice with tissue set aside for Hi-C sequencing. Head and thorax tissue was disrupted using a Nippi Powermasher fitted with a BioMasher pestle. High molecular weight (HMW) DNA was extracted using the Qiagen MagAttract HMW DNA extraction kit. HMW DNA was sheared into an average fragment size of 12–20 kb in a Megaruptor 3 system with speed setting 30. Sheared DNA was purified by solid-phase reversible immobilisation using AMPure PB beads with a 1.8X ratio of beads to sample to remove the shorter fragments and concentrate the DNA sample. The concentration of the sheared and purified DNA was assessed using a Nanodrop spectrophotometer and Qubit Fluorometer and Qubit dsDNA High Sensitivity Assay kit. Fragment size distribution was evaluated by running the sample on the FemtoPulse system.

### Sequencing

Pacific Biosciences HiFi circular consensus DNA sequencing libraries were constructed according to the manufacturers’ instructions. DNA sequencing was performed by the Scientific Operations core at the WSI on a Pacific Biosciences SEQUEL II (HiFi) instrument. Hi-C data were also generated from tissue of ilDreFalc1 using the Arima v2 kit and sequenced on the Illumina NovaSeq 6000 instrument.

### Genome assembly

Assembly was carried out with Hifiasm ( Cheng *et al.*, 2021) and haplotypic duplication was identified and removed with purge_dups ( Guan *et al.*, 2020). The assembly was then scaffolded with Hi-C data ( Rao *et al.*, 2014) using YaHS ( Zhou *et al.*, 2023). The assembly was checked for contamination and corrected as described previously ( Howe *et al.*, 2021). Manual curation was performed using HiGlass ( Kerpedjiev *et al.*, 2018) and Pretext ( Harry, 2022). The mitochondrial genome was assembled using MitoHiFi ( Uliano-Silva *et al.*, 2022), which performed annotation using MitoFinder ( Allio *et al.*, 2020). The genome was analysed and BUSCO scores generated within the BlobToolKit environment ( Challis *et al.*, 2020). Table 3 contains a list of all software tool versions used, where appropriate.

**Table 3.** Software tools and versions used.

Software tool	Version	Source
BlobToolKit	4.0.7	Challis *et al.*, 2020
Hifiasm	0.16.1-r375	Cheng *et al.*, 2021
HiGlass	1.11.6	Kerpedjiev *et al.*, 2018
MitoHiFi	2	Uliano-Silva *et al.*, 2022
PretextView	0.2	Harry, 2022
purge_dups	1.2.3	Guan *et al.*, 2020
YaHS	yahs-1.1.91eebc2	Zhou *et al.*, 2023

### Ethics and compliance issues

The materials that have contributed to this genome note have been supplied by a Darwin Tree of Life Partner. The submission of materials by a Darwin Tree of Life Partner is subject to the Darwin Tree of Life Project Sampling Code of Practice. By agreeing with and signing up to the Sampling Code of Practice, the Darwin Tree of Life Partner agrees they will meet the legal and ethical requirements and standards set out within this document in respect of all samples acquired for, and supplied to, the Darwin Tree of Life Project. All efforts are undertaken to minimise the suffering of animals used for sequencing. Each transfer of samples is further undertaken according to a Research Collaboration Agreement or Material Transfer Agreement entered into by the Darwin Tree of Life Partner, Genome Research Limited (operating as the Wellcome Sanger Institute), and in some circumstances other Darwin Tree of Life collaborators.

## Data Availability

European Nucleotide Archive: *Drepana falcataria* (pebble hook-tip). Accession number PRJEB54059; https://identifiers.org/ena.embl/PRJEB54059 ( Wellcome Sanger Institute, 2022) The genome sequence is released openly for reuse. The *Drepana falcataria* genome sequencing initiative is part of the Darwin Tree of Life (DToL) project. All raw sequence data and the assembly have been deposited in INSDC databases. The genome will be annotated using available RNA-Seq data and presented through the Ensembl pipeline at the European Bioinformatics Institute. Raw data and assembly accession identifiers are reported in Table 1.

## Author information

Members of the University of Oxford and Wytham Woods Genome Acquisition Lab are listed here: https://doi.org/10.5281/zenodo.4789928.

Members of the Darwin Tree of Life Barcoding collective are listed here: https://doi.org/10.5281/zenodo.4893703.

Members of the Wellcome Sanger Institute Tree of Life programme are listed here: https://doi.org/10.5281/zenodo.4783585.

Members of Wellcome Sanger Institute Scientific Operations: DNA Pipelines collective are listed here: https://doi.org/10.5281/zenodo.4790455.

Members of the Tree of Life Core Informatics collective are listed here: https://doi.org/10.5281/zenodo.5013541.

Members of the Darwin Tree of Life Consortium are listed here: https://doi.org/10.5281/zenodo.4783558.

## References

[ref-1] Allio R Schomaker-Bastos A Romiguier J : MitoFinder: Efficient automated large‐scale extraction of mitogenomic data in target enrichment phylogenomics. *Mol Ecol Resour.* 2020;20(4):892–905. 10.1111/1755-0998.13160 32243090 PMC7497042

[ref-2] Challis R Richards E Rajan J : BlobToolKit - interactive quality assessment of genome assemblies. *G3 (Bethesda).* 2020;10(4):1361–1374. 10.1534/g3.119.400908 32071071 PMC7144090

[ref-3] Cheng H Concepcion GT Feng X : Haplotype-resolved *de novo* assembly using phased assembly graphs with hifiasm. *Nat Methods.* 2021;18(2):170–175. 10.1038/s41592-020-01056-5 33526886 PMC7961889

[ref-4] GBIF Secretariat: *Drepana falcataria* (Linnaeus, 1758), GBIF Backbone Taxonomy. 2022; (Accessed: 25 February 2023). Reference Source

[ref-5] Guan D McCarthy SA Wood J : Identifying and removing haplotypic duplication in primary genome assemblies. *Bioinformatics.* 2020;36(9):2896–2898. 10.1093/bioinformatics/btaa025 31971576 PMC7203741

[ref-6] Harry E : PretextView (Paired REad TEXTure Viewer): A desktop application for viewing pretext contact maps. 2022; (Accessed: 19 October 2022). Reference Source

[ref-7] Henwood B Sterling P Lewington R : Field Guide to the Caterpillars of Great Britain and Ireland.London: Bloomsbury,2020. Reference Source

[ref-8] Howe K Chow W Collins J : Significantly improving the quality of genome assemblies through curation. *GigaScience.* Oxford University Press,2021;10(1):giaa153. 10.1093/gigascience/giaa153 33420778 PMC7794651

[ref-9] Kerpedjiev P Abdennur N Lekschas F : HiGlass: Web-based visual exploration and analysis of genome interaction maps. *Genome Biol.* 2018;19(1):125. 10.1186/s13059-018-1486-1 30143029 PMC6109259

[ref-10] Manni M Berkeley MR Seppey M : BUSCO Update: Novel and Streamlined Workflows along with Broader and Deeper Phylogenetic Coverage for Scoring of Eukaryotic, Prokaryotic, and Viral Genomes. *Mol Biol Evol.* 2021;38(10):4647–4654. 10.1093/molbev/msab199 34320186 PMC8476166

[ref-11] Randle Z Evans-Hill LJ Parsons MS : Atlas of Britain & Ireland’s Larger Moths.Newbury: NatureBureau,2019. Reference Source

[ref-12] Rao SSP Huntley MH Durand NC : A 3D map of the human genome at kilobase resolution reveals principles of chromatin looping. *Cell.* 2014;159(7):1665–80. 10.1016/j.cell.2014.11.021 25497547 PMC5635824

[ref-13] Rhie A McCarthy SA Fedrigo O : Towards complete and error-free genome assemblies of all vertebrate species. *Nature.* 2021;592(7856):737–746. 10.1038/s41586-021-03451-0 33911273 PMC8081667

[ref-14] Uliano-Silva M Ferreira JGRN Krasheninnikova K : MitoHiFi: a python pipeline for mitochondrial genome assembly from PacBio High Fidelity reads. *bioRxiv.* [Preprint],2022. 10.1101/2022.12.23.521667 PMC10354987 37464285

[ref-15] Waring P Townsend M Lewington R : Field Guide to the Moths of Great Britain and Ireland: Third Edition.Bloomsbury Wildlife Guides,2017. Reference Source

[ref-17] Wellcome Sanger Institute: The genome sequence of the Pebble Hook-tip, *Drepana falcataria* (Linnaeus, 1758). European Nucleotide Archive.[dataset], accession number PRJEB54059,2022.

[ref-16] Zhou C McCarthy SA Durbin R : YaHS: yet another Hi-C scaffolding tool. *Bioinformatics.* Edited by C. Alkan,2023;39(1):btac808. 10.1093/bioinformatics/btac808 36525368 PMC9848053

