# Supplementary material for: The genome sequence of the Pebble Hook-tip, Drepana falcataria (Linnaeus, 1758)
Source: Wellcome Open Res. 2023 May 10;8:207. [Version 1] doi: 10.12688/wellcomeopenres.19213.1 (PMC11101918; doi:10.12688/wellcomeopenres.19213.1)
Supplement: Peer review history

# Reviewer report: Reviewer response for version 1

**DOI:** 10.21956/wellcomeopenres.21295.r77118

**Published:** 2024-07-16

**Authors:** Joe I. Martinez

**Affiliations:** 1 University of Florida, Florida, USA

Boyes and Lewis present a genome assembly of the Pebble Hook-tip, *Drepana falcataria* (Linnaeus, 1758), which demonstrates a high degree of completeness, as evidenced by the elevated BUSCO score. The assembly is organized into 31 chromosomal pseudomolecules, including the assembled Z sex chromosome. This, combined with other well-described methodologies, has produced high-quality genomic data.

 The rationale behind creating the dataset is standard, with clear and easily understandable descriptions, making it straightforward to follow. The protocols and procedures are appropriate, and the methods and materials are presented in a manner that facilitates replication. Ultimately, the dataset is highly standardized, accompanied by comprehensive illustrations, and easily accessible, rendering it exceptionally valuable for various applications.

 Additionally, the quality of the genome can be enhanced through manual adjustments, making it a valuable reference for studying the systematics and evolution of the Drepanidae family. It can also facilitate the annotation of important genes associated with these groups of moths. This is particularly crucial considering that the family Drepanidae is among the least studied groups of moths.

Are sufficient details of methods and materials provided to allow replication by others?

Yes

Is the rationale for creating the dataset(s) clearly described?

Yes

Are the datasets clearly presented in a useable and accessible format?

Yes

Are the protocols appropriate and is the work technically sound?

Yes

Reviewer Expertise:

Evolutionary Biology, Ecology, Integrative Biology, Entomology

I confirm that I have read this submission and believe that I have an appropriate level of expertise to confirm that it is of an acceptable scientific standard.

## References

[1] : A pilot study on the molecular phylogeny of Drepanoidea (Insecta: Lepidoptera) inferred from the nuclear gene EF-1alpha and the mitochondrial gene COI. *Bull Entomol Res* .2010;100(2) : 10.1017/S0007485309990162 207-16 10.1017/S0007485309990162 19580687

# Reviewer report: Reviewer response for version 1

**DOI:** 10.21956/wellcomeopenres.21295.r72031

**Published:** 2024-05-25

**Authors:** Michael Hiller

**Affiliations:** 1 LOEWE-Centre for Translational Biodiversity Genomics (TBG), Senckenberg Nature Research Society, Frankfurt Am Main, Germany

This data note reports another high quality genome assembly of a lepidopteran species. All method, including version numbers of the software tools are clearly described, and the genome will certainly be valuable for lepidopteran comparative genomics.

 I only have one question on why a male (the homogametic sex, lacking chr W) was picked for sequencing? If this species is common, was it not possible to pick a female?

Are sufficient details of methods and materials provided to allow replication by others?

Yes

Is the rationale for creating the dataset(s) clearly described?

Yes

Are the datasets clearly presented in a useable and accessible format?

Yes

Are the protocols appropriate and is the work technically sound?

Yes

Reviewer Expertise:

genome assembly, comparative genomics

I confirm that I have read this submission and believe that I have an appropriate level of expertise to confirm that it is of an acceptable scientific standard.

# Reviewer report: Reviewer response for version 1

**DOI:** 10.21956/wellcomeopenres.21295.r81072

**Published:** 2024-05-17

**Authors:** Leonardo Barbosa Koerich

**Affiliations:** 1 Federal University of Minas Gerais, State of Minas Gerais, Brazil

I find the report of Boyes and Lewis, entitled: "The genome sequence of the Pebble Hook-tip, Drepana falcataria (Linnaeus, 1758)" to be of high quality. In the study, the authors present the genome assembly of the Peeble-hook-tip ( *Drepana falcataria*), assembled in 31 scaffolds at chromosome level.

 The genome sequencing process, outlined in detail, demonstrates a rigorous and well-executed approach. The use of Pacific Biosciences single-molecule HiFi long reads, complemented by chromosome conformation Hi-C data for scaffolding, ensures high-quality assembly. The manual curation efforts to correct misjoins further enhance the reliability of the assembly. The assembly statistics, including total length, scaffold N50, and chromosomal-level scaffolds, indicate a robust and comprehensive genome assembly. The high BUSCO completeness score validates the quality of the assembly, reinforcing its suitability for downstream analyses.

 As an overall assessment, the rationale for the study is clearly articulated, the methodologies are appropriate and well-described, and the datasets are presented in a clear and accessible manner.

Are sufficient details of methods and materials provided to allow replication by others?

Yes

Is the rationale for creating the dataset(s) clearly described?

Yes

Are the datasets clearly presented in a useable and accessible format?

Yes

Are the protocols appropriate and is the work technically sound?

Yes

Reviewer Expertise:

Genomics of insects vectors of diseases

I confirm that I have read this submission and believe that I have an appropriate level of expertise to confirm that it is of an acceptable scientific standard.

# Reviewer report: Reviewer response for version 1

**DOI:** 10.21956/wellcomeopenres.21295.r82209

**Published:** 2024-05-16

**Authors:** Kay Lucek

**Affiliations:** 1 University of Neuchâtel, Neuchâtel, Switzerland

The authors present the chromosome level genome assembly of a male specimen of the Pebble Hook-tip *Drepana falcataria*. The assembly consists of 31 chromosomes including the Z chromosome. The assembly is highly complete as revealed by the high BUSCO score but not fully phased. Sequencing and genome assembly follow the current state of the art and use established methods.

 Overall, the presented assembly will be of great value to study genome architecture as well as genome size evolution in Lepidoptera. However, as with other genome notes of the Darwin Tree of Life project, the genome is not yet annotated. Instead the data availability statement highlights that “The genome will be annotated using available RNA-Seq data and presented through the Ensemble pipeline at the European Bioinformatics Institute.” It would be important to update this genome note with the data identifier once the data is made public.

Are sufficient details of methods and materials provided to allow replication by others?

Yes

Is the rationale for creating the dataset(s) clearly described?

Yes

Are the datasets clearly presented in a useable and accessible format?

Yes

Are the protocols appropriate and is the work technically sound?

Yes

Reviewer Expertise:

Speciation Genomics

I confirm that I have read this submission and believe that I have an appropriate level of expertise to confirm that it is of an acceptable scientific standard.
